# Exploring the Relationship between the Structural Characteristics and Mechanical Behavior of Multicomponent Fe-Containing Phases: Experimental Studies and First-Principles Calculations

**DOI:** 10.3390/molecules28207141

**Published:** 2023-10-17

**Authors:** Dongtao Wang, Xiaozu Zhang, Hiromi Nagaumi, Minghe Zhang, Pengfei Zhou, Rui Wang, Bo Zhang

**Affiliations:** 1 High-Performance Metal Structural Materials Research Institute, Soochow University, Suzhou 215021, China; 2 School of Iron and Steel, Soochow University, Suzhou 215021, China; 3 School of Materials Science and Engineering, Liaoning University of Technology, Jinzhou 121001, China; 4 Shandong Weiqiao Aluminum & Electricity Co., Ltd., Binzhou 256200, China

**Keywords:** α-AlFeMnCrSi phase, stability, thermophysical properties, mechanical properties

## Abstract

A comprehensive understanding of the structural characteristics and mechanical behavior of Fe-containing phases is important for high-Fe-level Al-Si alloys. In this paper, the crystal characteristics, thermal stability, thermophysical properties and mechanical behavior of multicomponent α-AlFeMnSi and α-AlFeMnCrSi phases are investigated by experimental studies and first-principles calculations. The results indicate that it is easier for Fe and Cr to substitute the Mn-12j site in α-AlMnSi in thermodynamics; Cr is preferred to Fe for substituting Mn-12j/k sites due to its lower formation enthalpy after single substitutions at Mn atom sites. The α-AlFeMnCrSi phase shows higher thermal stability, modulus and intrinsic hardness and a lower volumetric thermal expansion coefficient at different temperatures due to the strong chemical bonding of Si-Fe and Si-Cr. Moreover, the α-AlFeMnCrSi phase has a higher ideal strength (10.65 GPa) and lower stacking fault energy (1.10 × 10^3^ mJ/m^2^). The stacking fault energy evolution of the different Fe-containing phases is mainly attributed to the differential charge-density redistribution. The strong chemical bonds of Si-Fe, Si-Mn and Si-Cr are important factors affecting the thermophysical and mechanical behaviors of the α-AlFeMnCrSi phase.

## 1. Introduction

Fe-containing phases such as β-AlFeSi, α-AlFeSi, α-AlFeMnSi and α-AlFeMnCrSi phases are common in Al-Si alloys and show their influence on the thermophysical and mechanical properties of Al-Si alloys [1,2,3,4,5,6,7,8,9]. The Fe-containing phases with different crystal structures and compositions show different thermophysical and mechanical properties. The addition of Mn resulted in the transformation of monoclinic β-Al_4.5_FeSi (A2/a) into cubic α-Al_19_Fe_4_MnSi_2_ (Im-3), and the precipitation temperature of quaternary Fe-containing phase increased after Mn addition [6,7,8,10,11]. After the co-addition of Mn and Cr, the monoclinic β-Al_4.5_FeSi phase transformed into an α-AlFeMnCrSi phase, which was ascertained to be a cubic crystal structure with complicated Fe/Mn/Cr atomic occupations; the α-AlFeMnCrSi phase showed a high precipitation temperature, high modulus and high intrinsic hardness [6,12,13,14].

The thermophysical and mechanical properties of intermetallics are derived from the characteristics of their crystal structures [7,8,15,16,17,18,19]. First-principles calculations based on the density functional theory (DFT) is an effective method for exploring the relationship between structural characteristics and properties. L. Amirkhanyan et al. reported the heat capacity and thermal expansion coefficient of τ4-Al_3_FeSi_2_ by first-principles calculations; the calculated heat capacity values were well validated by DSC (differential scanning calorimetry) measurements [17]. Dinsdale et al. reported the energetics and structures of Al_13_Fe_4_ and α-AlFeMnSi by first-principles calculations. It was found that Fe and Mn mixing on the crystallographic sites resulted in a negative mixing enthalpy, and the thermal stability of the α-AlFeMnSi phase improved [16]. Using first-principles calculations, Zhang et al. reported that the improved precipitation temperature of α-AlFeMnSi was attributed to the enhanced stability and strengthened chemical bonding of Si-Mn bonds [7,8].

In our previous studies, the evolution of the mechanical modulus and hardness (at 0 K) in β-Al_4.5_FeSi, α-AlMnSi and α-AlFeMnSi phases was discussed by analyzing electronic structures, chemical bonding, bond population and the electron density difference distribution calculation [7,8]. Many research studies also examined the thermophysical and mechanical properties of Fe-containing phases at 0 K [15,16,17,18]. However, their mechanical properties at elevated temperatures and mechanical behavior during the deformation of Fe-containing phases, including their mechanical modulus and hardness at elevated temperatures, ideal tensile strength and stacking fault energy during deformation, are also important for their engineering applications. These important properties have not been investigated in detail, and the relationship between structural characteristics and these properties is not clear. Moreover, in α-AlFeMnSi or α-AlFeMnCrSi phases, the Fe, Mn and Cr atoms can substitute for each other in the crystal structures due to their similar atomic radii and electrochemical properties [16,20,21,22]. Their preferential Wyckoff sites may affect the mechanical properties as mentioned above, which should also be investigated in detail.

In this paper, the structural characteristics, mechanical properties and behavior of the multicomponent α-AlFeMnSi and α-AlFeMnCrSi phases are investigated by experimental studies and first-principles calculations. The temperature-dependent heat capacity, thermal expansion coefficient, mechanical modulus and hardness at elevated temperatures, ideal tensile strength and stacking fault energy during deformation were studied to provide data support in the thermodynamic modelling and engineering application of high-Fe-level Al-Si alloys.

## 2. Experimental Method and Calculation Details

### 2.1. Experimental Method

The experimental alloys were prepared from pure Al (99.95%), Al-20%Si (all in mass fractions hereafter, unless otherwise specified), Al-10%Mn, A1-20%Fe and Al-10%Cr master alloys. These alloys were referred to as A1 and A2, respectively. The experimental chemical compositions of the as-prepared alloys were measured by a direct-reading spectrometer (Spectrometer LAB) and shown in Table 1. The experimental alloys were melted at 750 °C for 40 min in an electromagnetic induction furnace, and then the alloys were transferred into a holding furnace with a pre-set temperature of 660 °C for 60 min. During the holding process, the Fe-containing phases can precipitate and sedimentate to the bottom of the crucible due to their higher density [22]. After the holding process, the melt was slowly cooled at a cooling rate of 4 °C/min, and the bottom alloy was taken for observation and composition measurement of the Fe-containing phases.

The melting temperatures of the Fe-containing intermetallics were determined by a DSC (NETZSCH DSC204) with a heating process of 10 °C/min. The microstructures of Fe-containing phases were characterized by scanning electron microscopy (SEM, phenom XI), and their compositions were determined by an energy-dispersive spectrometer (EDS) equipped with SEM. Samples for scanning electron microscopy were mechanically ground and polished down with an OP-U (Oxide Polishing-Umber) colloidal silica suspension. The crystalline structures of the Fe-containing phases were analyzed by X-ray diffraction (XRD, Ultima Ⅳ), using Cu Kα (λ = 0.1540598 nm) radiation at a scanning rate of 10°/min. The TEM images and selected area electron diffraction (SAED) of the Fe-containing phases were characterized by transmission electron microscopy (TEM, JEM-2100). The samples for TEM were processed by an ion-thinning apparatus (Gatan 691) to guarantee that the thickness was less than 10 nm. Nanoindentation technology was performed on an iNano instrument (iMicro, Milpitas, CA, USA) to characterize the mechanical properties of the α-AlFeMnSi and α-AlFeMnCrSi phases with a force of 180 mN and the same dwell time of 10 s for all temperatures. The Young’s modulus and hardness values were derived from the load–displacement curves under loading/unloading through appropriate data analyses. Hot-stage tests were carried out on individually selected particles of the α-AlFeMnSi and α-AlFeMnCrSi phases at elevated temperatures (300, 400, 500 and 600 K). The experiments were performed in a box under nitrogen atmosphere to maintain stable environmental conditions for the hot stage. Simultaneously, oxidation effects on the surface of the samples should be minimized.

### 2.2. Calculation Details

In this study, all calculations were performed using the Cambridge Serial Total Energy Package codes [23]. To ensure that the calculations converged for all calculation accuracies, the maximum cutoff energy of the plane wave for all these phases was 450 eV, and 3 × 3× 3 k points in the first Brillouin zone were employed for all structures. The interaction between the valence electron and the ionic solid is adopted by the ultra-soft pseudopotentials [24]. The 3s^2^3p^1^, 3s^2^3p^2^, 3d^5^4s^2^, 3d^6^4s^2^ and 3d^5^4s^1^ were treated as valence electron configurations for Al, Si, Mn, Fe and Cr atoms, respectively. The exchange–correlation functional was described by the generalized gradient approximation with Perdew–Burke–Ernzerhof for surfaces and solids (PBEsol) [25]. The process of geometric optimization was carried out using the Broyden–Fletcher–Goldfarb–Shanno algorithm [26]. The convergence accuracy of the total energy, force, stress and displacement was less than 1 × 10^−5^ eV/atom, 0.001 eV/Å, 0.05 GPa, and 0.001 Å, respectively. The phonon spectrum and phonon density of states were calculated with the finite displacement method; this method was used to construct a supercell, move the atom, and calculate the force of all the atoms in the cell. A force-constant matrix was constructed based on this force, and the cutoff radius of the finite displace method was 5.0 Å. All DFT calculations were performed for seven volumes around the 0 K equilibrium volume of all structures to promote the quasi-harmonic approximation (QHA), and the elastic constants and mechanical properties under finite temperature were adapted using the quasi-static approximation (QSA) method [27,28].

#### 2.2.1. Thermodynamic Properties

Thermodynamic properties are very important, as they play an important role in understanding the service performance of these strengthened phases. To account for the finite temperature properties of a crystal, the quasi-harmonic Debye model [29] was applied in this study, such that the vibrational heat capacity is given by

(1) $$C_{V,vib}=3nk\left[4D\left(\Theta /T\right)-\frac{3\Theta /T}{e^{\Theta /T}-1}\right]$$

where C*_V_*_,*vib*_ is the vibrational heat capacity, *n* is the number of atoms per formula unit, *Θ* is the Debye temperature, and *k* is the Boltzmann constant. The thermal electronic contribution to the heat capacity can be calculated by

(2) CelV,T=∂Eel(V,T)∂T

where *E_el_* (*V*,*T*) is the thermal electron energy, which can be calculated by

(3) $$E_{el}\left(V,T\right)=\int n\left(\varepsilon ,V\right)f\left(\varepsilon ,\;V,T\right)\varepsilon d\varepsilon -\int \varepsilon^{F}n(\varepsilon ,V)\varepsilon d\varepsilon$$

where *f* is the Fermi function, *n*(*ε*,*V*) is the electronic density of states, and *ε* is the one-electron band energy. The isobaric heat capacity (*C_p_*) at constant volume can be calculated by

(4) $$C_{p}=C_{V}+VTB_{T}{\left(\beta (T)\right)}^{2}$$

(5) $$C_{V}=C_{V,vib}+C_{el}$$

where *β*(*T*) and *V* are the volumetric thermal expansion coefficients (TECs) and equilibrium volume, respectively. The *β*(*T*) values are obtained according to the equilibrium volume by employing the EOS method, as described in the following equation [29]:

(6) βT=dV(T)V(T)dT

#### 2.2.2. Mechanical Modulus

To calculate the mechanical modulus, the projected cubic elastic constants were obtained as averages following the method described in [29,30]:

(7) $${\bar{C}}_{11}=\left(C_{11}+C_{22}+C_{33}\right)/3$$

(8) $${\bar{C}}_{12}=\left(C_{12}+C_{13}+C_{23}\right)/3$$

(9) $${\bar{C}}_{44}=\left(C_{44}+C_{55}+C_{66}\right)/3$$

The shear and bulk modulus based on the elastic constant were calculated by the following equations for these intermetallics [31]:

(10) $$B_{V}=B_{R}=\left({\bar{C}}_{11}+{2\bar{C}}_{12}\right)/3$$

(11) $$G_{V}=\left({\bar{C}}_{11}-{\bar{C}}_{12}+3{\bar{C}}_{44}\right)/5$$

(12) $$G_{R}={5\left({\bar{C}}_{11}-{\bar{C}}_{12}\right)\bar{C}}_{44}/[4{\bar{C}}_{44}+3\left({\bar{C}}_{11}-{\bar{C}}_{12}\right)]$$

where $\bar{C}$_ij_ are the elastic constants, and the positions of $\bar{C}$_ij_ in elastic matrix are determined by i and j. Due to the high symmetry of the cubic structure, $\bar{C}$_11_, $\bar{C}$_12_ and $\bar{C}$_44_ are selected as independent elastic constants for the mechanical properties calculation. *B_V_* (*B_R_*) and *G_V_* (*G_R_*) are the bulk modulus and shear modulus, respectively, calculated from the Voigt (Reuss) model; moduli values obtained from the Voigt model are usually larger than those from the Reuss model, and their average value is suggested as a good approximation [32]. Therefore, the final *B* and *G* values can be acquired by calculating their average values [33]:

(13) $$B=(B_{V}+B_{R})/2$$

(14) $$G=(G_{V}+G_{R})/2$$

The Young’s modulus (*E*) can be evaluated as follows:

(15) E=9BG/(3B+G)

In this work, two different models were applied to predict the temperature-dependent hardness of the α-AlFeMnSi and α-AlFeMnCrSi phases, which are expressed as Chen’s model (*H_VC_*) and Tian’s model (*H_VT_*), respectively [34].

(16) $$H_{VC}=2(k^{-2}G{)}^{0.585}-3$$

(17) $$H_{VT}=0.92k^{-1.137}G^{0.708}$$

where *k* is the Pugh ratio; *k* = B/G.

#### 2.2.3. Stacking Fault Energy

The supercell model of the α-AlMnSi, α-AlFeMnSi and α-AlFeMnCrSi phases must fully relax to eliminate stress before performing the first-principles tensile test experiment, and the normal strain occurs along the z-direction, while the x- and y- directions are fixed. The ideal tensile strengths of the α-AlMnSi, α-AlFeMnSi and α-AlFeMnCrSi phases are obtained after the interfacial breakage, and the normal strain is described as [35]:

*ε*_tensile_ = (*l* − *l*_0_)/*l*_0_ (18)

In this formula, *l*_0_ and *l* are the initial cell length and the cell length after deformation, respectively. The stacking fault is the energy penalty between a perfect crystallographic plane and a crystallographic plane after shear deformation. The stacking fault energy is produced in a crystal when a local deviation occurs in the stacking sequence [36]. It can be defined as [37]:

γ(b) = (E(b) − E_0_)/A (19)

where E_0_ is the energy of the perfect structure, E(b) is the energy of the sheared lattice, and A is the area of the supercell basal plane. Shear strain is described as

*γ_shear_* = Δ*x*/*y* (20)

where Δ*x* and *y* are the shear deformation length and the initial cell length along the deformation direction, respectively. In this work, the engineering strain and the shear strain are increased by a step of 2% for all the supercell models with a quasi-static method [35].

## 3. Results and Discussion

### 3.1. Characteristics of Fe-Containing Phases by Experimental Studies

**Figure 1:**
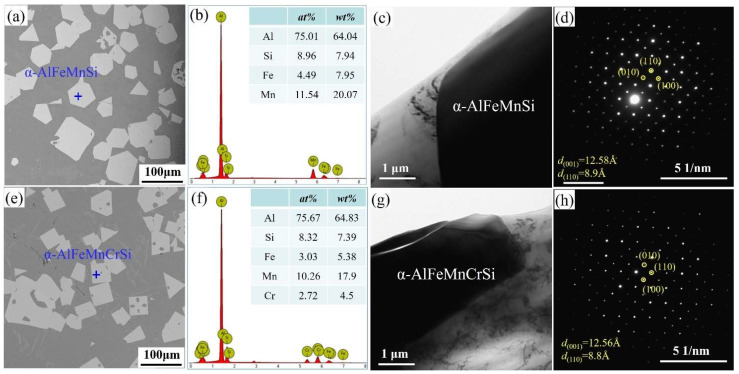
(**a**) SEM image, (**b**) EDS result, (**c**) TEM image and (**d**) selected area electron diffraction (SAED) of α-AlFeMnSi; (**e**) SEM image, (**f**) EDS result, (**g**) TEM image and (**h**) SAED of α-AlFeMnCrSi.

Figure 1 shows the morphologies, EDS and TEM results of Fe-containing phases in A1 and A2 alloys taken from the crucible bottom. The Fe-containing phases show a regular polygonal morphology (Figure 1a,e), and their compositions are shown in Figure 1b,f, respectively. The α-AlFeMnSi phase consists of 75.01 at.% Al, 4.49 at.% Fe, 11.54 at.% Mn and 8.96 at.% Si (Figure 1b). The α-AlFeMnCrSi phase consists of 75.67 at.% Al, 8.32 at.% Si, 3.03 at.% Fe, 10.26 at.% Mn and 2.72 at.% Cr (Figure 1f). In the A1 alloy, the atomic ratio of the α-AlFeMnSi phase differs from that of Al_19_Fe_4_MnSi_2_ (ICSD#655126) because the atomic ratio is not a constant value in Al-Si-Fe-Mn-Cr alloys with different mass fractions. In this paper, the chemical formula is ascertained as Al_75.01_(Fe_4.49_Mn_11.54_)Si_8.96_ and Al_75.67_(Fe_3.03_Mn_10.26_Cr_2.72_)Si_8.32_ for the α-AlFeMnSi and α-AlFeMnCrSi phases, according to the EDS results. These results show that the atomic percents of (Al + Si) in α-AlFeMnSi and α-AlFeMnCrSi change in a small range. The atomic percents of (Fe + Mn) and (Fe + Mn + Cr) also change in a very small range. Tibballs et al. studied the α-AlFeMnSi phase with different Fe levels and found that an Fe atom could substitute for a Mn atom in the unit cell in the α-AlFeMnSi phase [11]. Simensen et al. investigated the α-AlFeMnSi phase with different contents of Mn, Fe and Si and found that Fe could substitute for Mn + Si atoms in the unit cell [38]. The composition change in α-AlFeMnCrSi indicates the substitution of Mn or Fe atoms by Cr, which is in accordance with our previous study, in which α-AlFeMnSi and α-AlFeMnCrSi phases were formed by replacing part of the Mn atoms in α-AlMnSi with Fe or Fe + Cr respectively [7,22].

To further identify the crystal structures of the α-AlMnFeSi and α-AlFeMnCrSi phases in this study, all of these were examined by TEM. The TEM images and SAEDs of α-AlMnFeSi and α-AlFeMnCrSi along the [001] zone axis are shown in Figure 1c,d,g,h. The SAED patterns indicate that α-AlMnFeSi and α-AlFeMnCrSi are cubic structures; the lattice parameters of the α-AlMnFeSi and α-AlFeMnCrSi phases are 12.58 and 12.56 ± 0.01 Å, respectively, which show some deviation to the reported values of 12.7 Å and 12.56 Å of α-AlMnFeSi in the literature [38]. This tiny deviation may be caused by the atomic radius difference of Fe, Mn and Cr or by the solid solubility floating of some atoms in these phases [7,11,22,38,39]. The interplanar spacings on the (110) plane are 0.89 nm for the α-AlMnFeSi phase and 0.88 nm for the α-AlFeMnCrSi phase.

**Figure 2:**
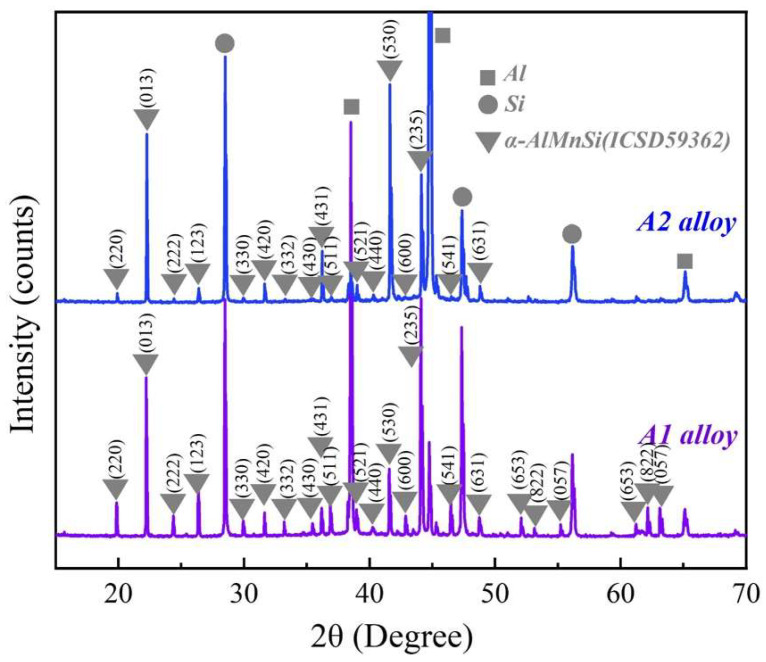
XRD patterns of the A1 and A2 alloys.

Figure 2 shows the XRD patterns of the A1 and A2 alloys. Except for diffraction peaks of the Al and Si phases, the main diffraction peaks match the diffraction peaks of the α-AlMnSi phase (ICSD#59362) at different (*h k l*) in the A1 and A2 alloys. The EDS results in Figure 1 indicate that the A1 and A2 alloys consist of Al, Si and Mn,Si-containing phases: the Mn,Si-containing phases are α-AlMnFeSi and α-AlFeMnCrSi in the A1 and A2 alloys, which imply that the α-AlMnFeSi and α-AlFeMnCrSi phases have the same crystal structure as the α-AlMnSi phase. The XRD results confirm the cubic structures of the α-AlMnFeSi and α-AlFeMnCrSi phases with the space group of Pm-3, and the refined lattice parameters of a = b = c = 12.65 Å. These results are consistent with the observation of the TEM images in Figure 1. The tiny deviation in lattice parameters may be caused by the tiny atom-fraction floating of Si, Fe, Mn and Cr atoms in the TEM observation positions. Combining the results in Figure 1, the doping of Fe or Cr in the α-AlMnSi phase does not change the cubic crystal structure; the Fe and Cr atoms substitute for the Mn atoms in the cubic crystal structure of the α-AlMnSi phase, resulting in the change in EDS results.

**Figure 3:**
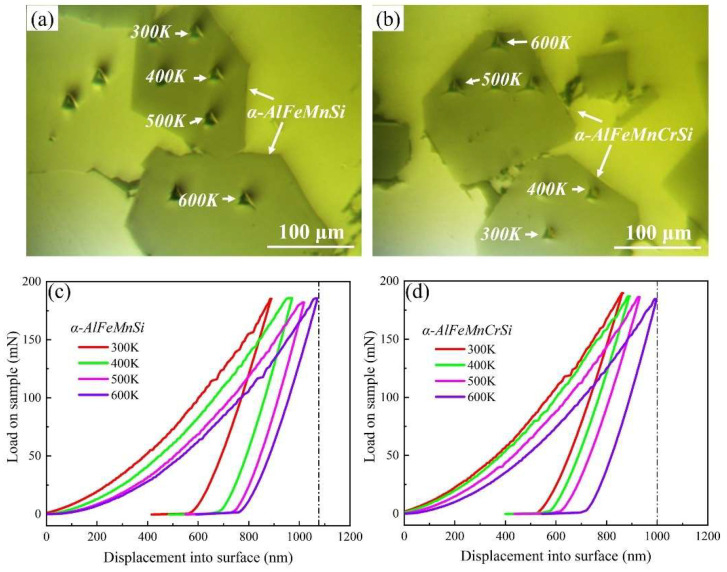
The indentation position and morphology on (**a**) α-AlFeMnSi and (**b**) α-AlFeMnCrSi. Typical load–displacement curves for (**c**) α-AlFeMnSi and (**d**) α-AlFeMnCrSi at different temperatures.

The nanoindentation measurements were performed to obtain the Young’s modulus and hardness of the α-AlFeMnSi and α-AlFeMnCrSi phases at ambient and elevated temperatures. The position and morphology of the indentations are shown in Figure 3a,b. The typical load–displacement curves and corresponding results are shown in Figure 3c,d and Table 2. Evidently, the higher hardness of the phases is visible in the smaller maximum indentation depth at peak loads. The Young’s modulus and hardness values of the α-AlFeMnSi phase at 300, 400, 500 and 600 K are 215.9, 205.2, 199.1, 192.6 GPa and 14.2, 13.3, 12.8, 11.9 GPa, respectively. After doping with Cr atoms, the Young’s modulus and hardness values of the α-AlFeMnCrSi phase improved to 224.8, 218.5, 210.6, 201.5 GPa and 16.98, 15.84, 14.7, 13.1 GPa at 300, 400, 500 and 600 K, respectively.

Chen et al. reported that the Young’s modulus and hardness of the α-AlFeMnSi phase were 175.3, 146.0, 132.7 GPa and 10.82, 10.08, 8.14 GPa, respectively, at room temperature, 200 °C and 350 °C, which are lower than those of the α-AlFeMnSi phase in this study due to the phase composition deviation [40]. Moreover, the Young’s modulus and hardness of the α-AlFeMnSi and α-AlFeMnCrSi phases at room and elevated temperature are higher than that of Si (175.3, 146.0, 132.7 GPa and 11.13, 11.12, 9.21 GPa at room temperature, 200 °C and 350 °C, respectively), Al_9_FeNi (161.5, 137.7 GPa and 7.71, 6.96 GPa at room temperature and 200 °C, respectively) and other Ni,Cu-containing intermetallics [40], indicating their high thermostability. The Young’s modulus and hardness of Fe-containing phases in this study show a decreasing tendency with increasing temperature.

### 3.2. Crystal Structure Models of DFT Calculations

**Figure 4:**
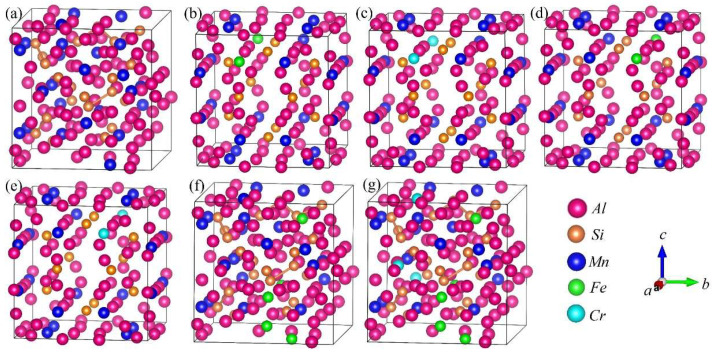
Crystal structure projections of Cr or Fe occupying the Mn atom sites in the α-AlMnSi phase. (**a**) α-AlMnSi; (**b**) α-AlMnFe_Mn-12j_Si; (**c**) α-AlMnCr_Mn-12j_Si; (**d**) α-AlMnFe_Mn-12k_Si; (**e**) α-AlMnCr_Mn-12k_Si; (**f**) α-AlFeMnSi; (**g**) α-AlFeMnCrSi. Fe_Mn-12j_ represents the Fe atom occupying the Mn-12j atom site in the α-AlMnSi phase.

DFT calculations were performed to analyze the formation enthalpy, energetically favored structure, chemical bond and properties of the α-AlFeMnSi and α-AlFeMnCrSi phases. The XRD results indicate that the α-AlMnSi phase has the same cubic structure as the α-AlFeMnSi and α-AlFeMnCrSi phases. Previous works show that Fe and Cr atoms can substitute for the Mn atom in the unit cell in the α-AlMnSi phase [7,22]. The α-AlMnSi phase (ICSD#59362) shows a cubic structure with *a* = *b* = *c* = 12.64 Å; there are a total of 136 atoms in the unit cell, and 24 Mn atoms occupy the 12j and 12k Wyckoff sites [41]. Detailed atom coordinates of Al, Mn and Si have been given in ref. [41]. Therefore, in this paper, the crystal models of the atom substitution at Mn sites by Fe and Cr are established, and the substituted atom coordinates of Mn-12j and Mn-12k are shown in Figure 4. The basic crystal structure of the α-AlMnSi phase is also analyzed to investigate the effect of Fe and Cr doping. The calculated crystal lattice constants and volume of the unit cell are shown in Table 3.

In order to determine the preferential sites of Fe and Cr in the α-AlMnSi crystal structure, the formation enthalpy of a single substitution at a Mn atom site (*E_f_*(*X_M_*)) is used to estimate the energetically favored configuration, as shown in Equation (21) [42].

(21) $$E_{f}\left(X_{M}\right)=Etotal\left(X_{M}\right)-Etotal(AlMnSi)-\sum n_{i}\mu_{i}$$

where *Etotal*(*AlMnSi*) and *Etotal*(*X_M_*) are the total energy of the perfect and defective supercell with a single defect *X_M_*, respectively. *n_i_* indicates the number of atoms that are added to (*n_i_* > 0) or removed from (*n_i_* < 0) the supercell when the defect structure is created.

The calculation models are handled with the special quasirandom structure (SQS) method implemented in the Alloy Theoretic Automated Toolkit (ATAT) software to obtain the most reasonable structure with different constituents [43,44].

The calculated formation enthalpies of Fe and Cr atoms substituting for Mn atoms are listed in Table 3. The formation enthalpy of Fe occupying Mn at the 12j site (−2.166eV/atom), as well as Cr (−2.802eV/atom), is remarkable lower than that of Fe and Cr substituting for Mn at the 12k sites (−1.795eV/atom and −2.346eV/atom). Therefore, Fe and Cr more easily substitute for Mn-12j in α-AlMnSi in thermodynamics. It also shows that Cr is preferred to Fe at both sites due to its lower formation enthalpy after single substitution at Mn atom sites.

The crystal structures of α-AlFeMnSi (Al_102_Mn_18_Fe_6_Si_12_) and α-AlFeMnCrSi (Al_102_Mn_14_Cr_4_Fe_6_Si_12_) with simplified atomic ratios were built according to the atomic ratio of Al_75.01_Fe_4.49_Mn_11.54_Si_8.96_ and Al_75.67_Fe_3.03_Mn_10.26_Cr_2.72_Si_8.32_ from the EDS results, and these are shown in Figure 4f,g. The calculated crystal lattice constants and volumes are shown in Table 3. In these crystal structures, Fe and Cr atoms first occupy the Mn-12j sites; then, the Fe and Cr atoms occupy the Mn-12k sites when the Mn-12j sites are fully occupied. The formation enthalpies (*E_f_*) in Table 3 show that α-AlFeMnCrSi (−3.351eV/atom) is thermodynamically more stable than α-AlFeMnSi (−2.722eV/atom) and α-AlMnSi (−1.2921eV/atom).

**Figure 5:**
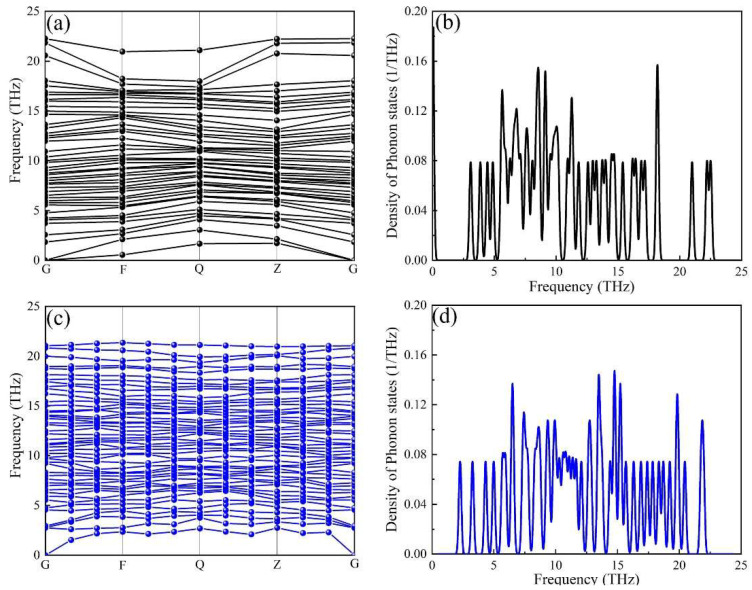
The calculated phonon dispersion curves of the (**a**) α-AlFeMnSi phase and (**c**) α-AlFeMnCrSi phase, and the corresponding phonon density of states for the (**b**) α-AlFeMnSi phase and the (**d**) α-AlFeMnCrSi phase.

The phonon dispersion curves and projected phonon densities of states (PHDOS) of the α-AlFeMnSi and the α-AlFeMnCrSi phases were calculated, and these are depicted in Figure 5. The absence of soft modes at any high-symmetry dispersion is indicated by the phonon calculations, which serve as a requirement for crystal stability along high-symmetry directions in the Brillouin zone. There is no imaginary frequency (frequency ≤ 0 THz) in the phonon dispersion curve, indicating that all the investigated phases are dynamically stable. The results prove that the substitution of Mn atoms in the α-AlMnSi phase by Fe and Cr is favorable in terms of dynamic stability.

### 3.3. Thermodynamic Properties and Mechanical Modulus

**Figure 6:**
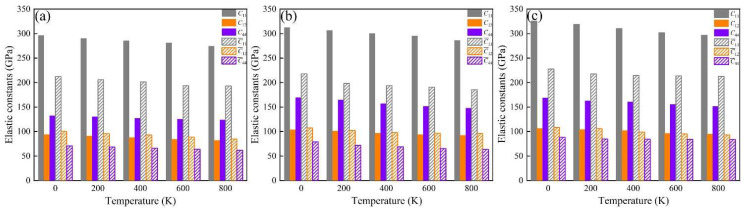
Temperature-dependent elastic constants of the (**a**) α-AlMnSi, (**b**) α-AlFeMnSi and (**c**) α-AlFeMnCrSi phases.

**Figure 7:**
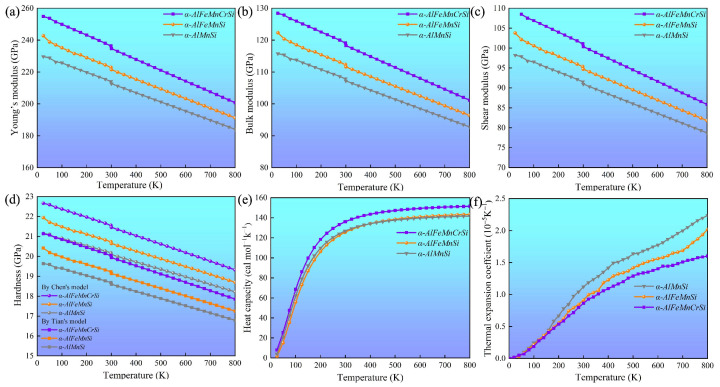
Temperature-dependent (**a**) Young’s modulus (*E*), (**b**) bulk modulus (*B*), (**c**) shear modulus (*G*), (**d**) hardness by Chen’s model and Tian’s model (*H_VC_* and *H_VT_*), (**e**) isobaric heat capacity and (**f**) volumetric thermal expansion coefficient of the α-AlMnSi, α-AlFeMnSi and α-AlFeMnCrSi phases.

The elastic constants (*C*_ij_, $\bar{C}$_ij_) of these phases at 0 K were calculated, and these are summarized in Table 4. These indicate that all the investigated phases obey the mechanical stability conditions based on the Born standard [42]. The calculated elastic constants of the different phases were obtained using the QSA method at different temperatures, as shown in Figure 6. The results show that the *C*_ij_ and $\bar{C}$_ij_ of the phases increase with Fe and Fe + Cr doping and decrease with an increase in temperature. The *C*_11_ and $\bar{C}$_11_ of the α-AlFeMnCrSi phase are larger than those of the other two phases. Moreover, the calculated temperature-dependent mechanical modulus, including Young’s (*E*), bulk (*B*) and shear (*G*) modulus, are depicted at different temperatures in Figure 7a–c. It indicates that the Young’s modulus value increases in the sequence of *E*_α-AlFeMnCrSi_ > *E*_α-AlFeMnSi_ > *E*_α-AlMnSi_ in each set of data at the same temperature, and that the shear and bulk moduli exhibit the same trend. The results indicate that the doping of α-AlMnSi with Fe can improve the mechanical modulus, and the doping with Cr and Fe in the α-AlMnSi phase can further improve the mechanical modulus and elastic heat-resistant property. The mechanical modulus for the α-AlMnSi, α-AlFeMnSi and α-AlFeMnCrSi phases decreases linearly with increasing temperature.

The intrinsic hardness is an important index for the mechanical evaluation of intermetallics [42]. The hardness of different phases, calculated by Chen’s model (*H*_VC_) and Tian’s model (*H*_VT_), indicates that the α-AlMnSi phase has the lowest hardness value, while the α-AlFeMnCrSi phase shows the highest hardness value in these three phases (Figure 7d), which shows a good agreement with the variation tendency in nanoindentation results. For all of these phases, the Young’s modulus and hardness values from the DFT calculations are larger than those from nanoindentation measurements at the same temperature. One reason for the discrepancy is that the actual chemical compositions of these phases are not fully consistent with our theoretical models. Another reason is that the nanoscale defects, surface roughness and surface contamination are not taken into consideration, which may lead to a large deviation of the test results [45].

Figure 7e shows that the isobaric heat capacity (*C_p_*) values of the α-AlMnSi, α-AlFeMnSi and α-AlFeMnCrSi phases increases dramatically at 0~300 K before increasing slowly at high temperatures (>300 K). The calculated *C_p_* values of the α-AlMnSi, α-AlFeMnSi and α-AlFeMnCrSi phases are approximately 126, 126 and 136 Cal/(mol·K) at room temperature, respectively. This indicates that the doping with Fe does not affect the *C_p_* of the phase, and that the doping of Fe and Cr improves the *C_p_* value. According to Equations (4) and (5), the isobaric heat capacity can be determined through the contributions of the lattice vibration, thermal electrons and thermal expansion. Moreover, the α-AlFeMnCrSi phase shows a lower thermal expansion (Figure 7f). Therefore, the contributions of lattice vibration may result in a higher *C_p_* value in the α-AlFeMnCrSi phase. The doping with Cr atoms may lead to a lower vibration frequency, resulting in the α-AlFeMnCrSi phase having a larger *C_p_* value.

The volumetric thermal expansion coefficients (TEC) of different phases are shown in Figure 7f. The TEC values of these phases decrease in the following sequence: TEC_α-AlMnSi_ > TEC_α-AlFeMnSi_ > TEC_α-AlFeMnCrSi_. The TEC value increases linearly, which is caused by the intensive phonon excitations and the enhancement of the anharmonic effect. It has been demonstrated that α-AlMnSi has a cubic structure with a chain of silicon rings linked by Si and Mn atoms, and the configuration of the silicon atoms is quite stable for the formation of covalent chemical bonds between them. After the substitution of Mn atoms by Fe or Cr, the chain of silicon rings linked by Si and Mn/Fe/Cr atoms leads to the formation of stronger Si-Mn/Fe/Cr covalent chemical bonds and slower increased TEC values at high temperature [46,47].

**Figure 8:**
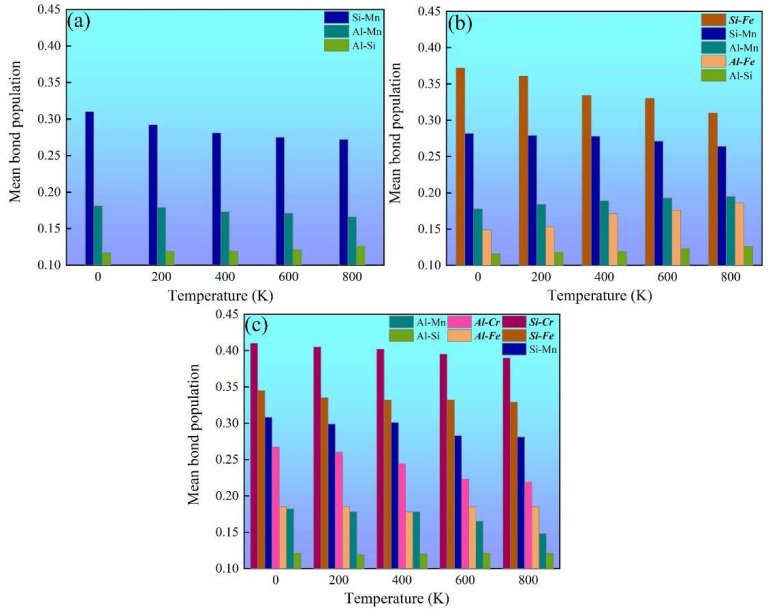
Mulliken population analysis for the temperature-dependent chemical bonds in (**a**) α-AlMnSi, (**b**) α-AlFeMnSi and (**c**) α-AlFeMnCrSi.

Chemical-bond strength is an important factor for the thermophysical and mechanical properties of multicomponent intermetallics such as the mechanical modulus and thermal expansion coefficient [29]. Mulliken’s population analysis is an effective method for estimating the chemical-bond-strength evolution [48,49,50,51]. The more positive the bond population, the stronger the chemical bonding [29,48,49,50,51]. The temperature-dependent mean bond populations of various chemical bonds in α-AlMnSi, α-AlFeMnSi and α-AlFeMnCrSi were calculated, and these are summarized in Figure 8. The Si-Mn bonds are the strongest chemical bonds in the α-AlMnSi phase due to their large bond-population value, which dominates the mechanical properties of the α-AlMnSi phase (Figure 8a). When Fe is doped into the α-AlMnSi phase, the Si-Fe and Si-Mn bonds dominate the mechanical properties of the α-AlFeMnSi phase, and the Si-Fe bond shows stronger chemical bonding (Figure 8b). After doping Fe and Cr into the α-AlMnSi phase, the high bond-population values of Si-Cr, Si-Fe and Si-Mn bonds show the dominant effect on the strengthening of mechanical properties and the decrease in thermal expansion (Figure 8c). Moreover, the Si-Cr bond population is larger than that of Si-Fe and Si-Mn bonds in the α-AlFeMnCrSi phase, which means the strongest chemical bonding of Si and Cr atoms. When the temperature increases, the mean bond-population values of dominant Si-Cr, Si-Fe, Si-Mn and Al-Cr bonds decreases, resulting in a decreased modulus and increased thermal expansion coefficients of different phases.

Table 5 shows the calculated mean bond lengths (Å) of the α-AlMnSi, α-AlFeMnSi and α-AlFeMnCrSi phases at different temperatures. For the α-AlMnSi phase, the length of the Si-Mn bond is 2.59 Å at 0 K, which is shorter than that of the Al-Si and Al-Mn bonds. It indicates that Si-Mn shows a stronger chemical bond. The doping of Fe into the α-AlMnSi phase results in the formation of a Si-Fe bond with a short bond length (2.52 Å at 0 K). When Fe and Cr are doped into the α-AlMnSi phase, the Si-Cr and Si-Fe bonds show a short bond length (2.56 and 2.65 Å at 0 K, respectively). The shorter chemical bond represents the stronger interaction between the atoms. In these α phases, these shorter chemical bonds show higher bond-population values (Figure 8), which proves that Si-Cr, Si-Fe and Si-Mn have strong chemical bonds. The results imply that the doping with Fe and Cr improves the thermostability and mechanical properties of the phases by the formation of strong Si-Fe/Cr chemical bonds.

**Figure 9:**
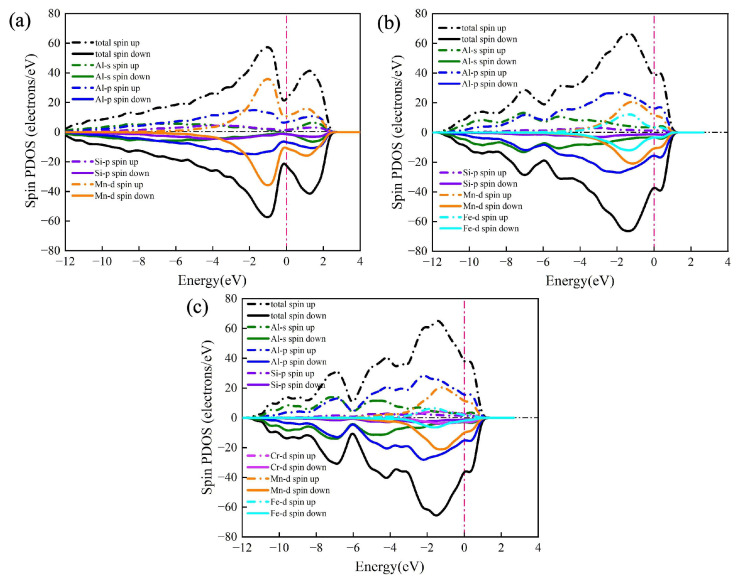
The density of states (DOS) of (**a**) α-AlMnSi, (**b**) α-AlFeMnSi and (**c**) α-AlFeMnCrSi. Dashed lines represent the Fermi level.

The total density of states (TDOS) and partial density of states (PDOS) of the α-AlMnSi, α-AlFeMnSi and α-AlFeMnCrSi phases are presented in Figure 9. The most obvious feature of these multicomponent phases is the metallic character because of the non-zero value of TDOS at the Fermi level. It is evident that the Mn-d orbital of the Mn atom determines the Fermi level of the α-AlMnSi phase in the −2 to 2 energy range due to the nearly identical evolution tendency and relatively approximate value of Mn-d’s PDOS and the TDOS of α-AlMnSi (Figure 9a). Meanwhile, the Fermi levels of the α-AlFeMnSi and the α-AlFeMnCrSi phases are determined by the Al-p and Mn-d orbitals simultaneously in the −2 to 2 energy range due to their larger PDOS value and similar evolution tendency with TDOS at this energy range (Figure 9b,c). Fermi levels are located at the shoulders of the peaks of TDOS for the α-AlMnSi, α-AlFeMnSi and α-AlFeMnCrSi phases, implying that all of them are stable. These results are consistent with the previous discussions on the formation enthalpy and phonon spectrum. Moreover, the thermal electronic contribution is determined by the electronic density of states near the Fermi energy, which does not show an obvious difference in the α-AlFeMnSi and α-AlFeMnCrSi phases (Figure 9).

For the α-AlMnSi phase, the hybridization of the Si-p and Mn-d bands is obvious due to the overlap of the Si-p and Mn-d bands in the −4 to −2 eV energy range, indicating the formation of Si-Mn chemical bonds at this energy range (Figure 9a). With the doping of Fe atoms into the α-AlMnSi phase, the TDOS is dominated by Al-p bands and Mn-d bands simultaneously (Figure 9b). The Si-p bands overlap with the Fe-d and Mn-d bands to form two different type chemical bonds in the −4 to −2 eV energy range, thus leading to an enhanced stability of the α-AlFeMnSi phase (Figure 9b). For the α-AlFeMnCrSi phase, the Fe-d band is weakened due to the substitution of Fe atoms by Cr. The Si-Mn, Si-Fe and Si-Cr chemical bonds form due to the hybridization of the Si-p and Mn-d, Fe-d, Cr-d bands in the −4 to −2 eV energy range (Figure 9c). These results also indicate that the evolution of Si-X bonds (X = Mn, Fe, Cr) is an important factor for the thermophysical and mechanical properties of multicomponent Fe-containing phases in Al-Si alloys.

### 3.4. Stacking Fault Energy and Tensile Properties

**Figure 10:**
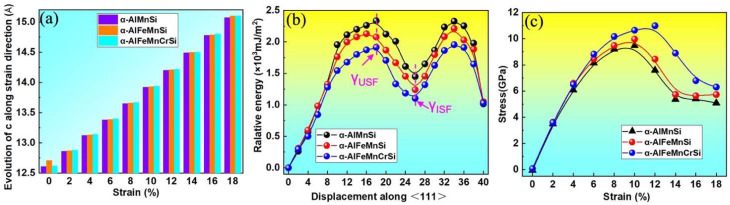
(**a**) The calculated lattice parameters of α-AlMnSi, α-AlFeMnSi and α-AlFeMnCrSi along the strain direction; (**b**) generalized stacking fault energies of {110}, [111] shear deformation and (**c**) stress–strain curves for the α-AlMnSi, α-AlFeMnSi and α-AlFeMnCrSi phases.

The stacking fault energy and tensile strength are critical mechanical property indicators of intermetallics. Stacking fault (SF) energies are known to govern the activation of various dislocation processes and deformation behavior of alloys and intermetallics [52]. To clarify the effects of Fe and Cr doping on the strength of the α-AlMnSi phase, their generalized stacking fault energy and ideal tensile strength are calculated by first-principles methods and shown in Figure 10. Figure 10a shows the length variation of the lattice constant along the c-axis for the α-AlMnSi, α-AlFeMnSi and α-AlFeMnCrSi phases. The unstable stacking fault energy (γ_USF_) and intrinsic stacking fault energy (γ_ISF_) are denoted in Figure 10b. The γ_ISF_ of the α-AlMnSi phase is 1.45 × 10^3^ mJ/m^2^, and the doping with Fe and Fe + Cr can decrease the γ_ISF_ to 1.24 × 10^3^ mJ/m^2^ for Fe doping and 1.10 × 10^3^ mJ/m^2^ for the combination doping with Fe + Cr. The results reveal that individual doping with Fe and combination doping with Fe + Cr decrease the stacking fault energy of the α-AlMnSi phase, which will motivate the activation of the slip systems [52].

The stress–strain curves of the α-AlMnSi, α-AlFeMnSi and α-AlFeMnCrSi phases are shown in Figure 10c. At the first stage (0~12%) of the tensile simulation for the α-AlFeMnCrSi phase, the stress increases gradually with the increase in strain and reaches the maximum of 10.65 GPa at a strain of 12%; then, the stress starts to decline, which implies the breaking of chemical bonds [50]. The maximum tensile stress values of the α-AlMnSi and α-AlFeMnSi phases are approximately 9.47 and 9.97 GPa at a strain of 10%. The α-AlFeMnCrSi phase has the highest stress, which may be related to the strong Si-Cr bonds.

**Figure 11:**
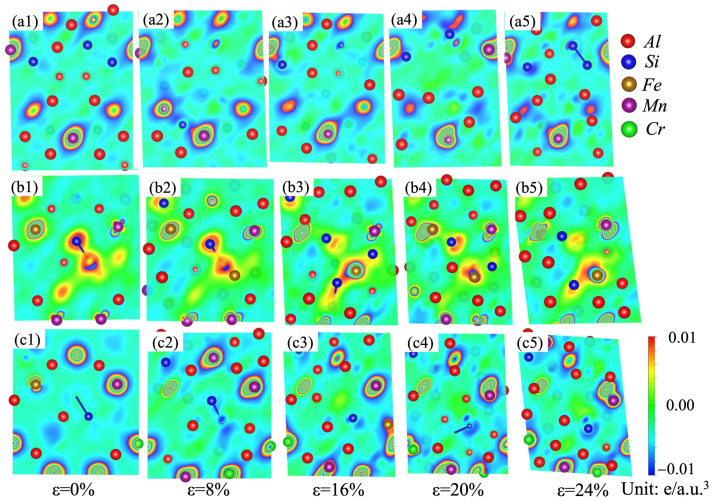
Charge-density difference on the (110) plane for the (**a1**–**a5**) α-AlMnSi; (**b1**–**b5**) α-AlFeMnSi; and (**c1**–**c5**) α-AlFeMnCrSi phases during shear strain.

Variations in the stacking fault energies and ideal tensile strengths can be analyzed in terms of the differential charge-density distributions [35,52,53,54,55]. A dense charge density usually means strong chemical bonding among the atoms. Nonspherical distribution of charge density after deformation retards the redistribution of the charge density, and thus hinders the shear deformation [35]. In order to understand the micro-mechanism and physical origin of the stacking fault energy evolution of the α-AlMnSi, α-AlFeMnSi and α-AlFeMnCrSi phases, the charge-density differences under different strains on the (110) plane are shown in Figure 11 to explore the bonding characteristics during tensile deformation. In Figure 11, the blue color stands for the loss of the electronic region, and the red color represents the acquisition of the electronic region.

Figure 11(a1–a5) show the differential charge density of the α-AlMnSi phase at different shear strains. It indicates that the areas between Mn and Al/Si atoms are filled with a large number of charges, which means that Mn can form much stronger bonds with Al/Si than Al–Al/Si bonds [54]. With the doping of Fe atoms into the α-AlMnSi phase, Fe–Al/Si bonds replace Mn–Al/Si bonds, and Fe-Si bonds show a stronger bonding strength due to the denser charge density around the Si atoms. At the first stage, the differential charge density for the α-AlMnSi phase with a 0% shear strain displays a cylindrical shape surrounding the Mn atomic position (Figure 11(a1)). After shear deformation (ε = 8% and 16%), the differential charge densities around the Mn atoms close to the stacking fault plane are redistributed to fit the stacking fault configuration, whereas the differential charge densities around Al and Si atoms are affected less (Figure 11(a2–a5)). When the shear strains on the α-AlMnSi phase are 20% and 24%, the charge distribution around the Mn atoms in the α-AlMnSi phase shows a decreasing tendency. For the α-AlFeMnSi phase, the charge density around the Mn atoms is lower than that in the α-AlMnSi phase, which leads to a decreased redistribution resistance and a lower stacking fault energy. The charge distribution around the Cr atoms determines the stacking fault energy evolution in the α-AlFeMnCrSi phase. The lower charge distribution around the Cr atoms means less shear-strain resistance and lower stacking fault energy. It indicates that the stacking fault energy evolution of the α-AlMnSi, α-AlFeMnSi and α-AlFeMnCrSi phases is mainly attributed to the differential charge-density redistribution (such as the nonspherical shape and magnitude) [35].

**Figure 12:**
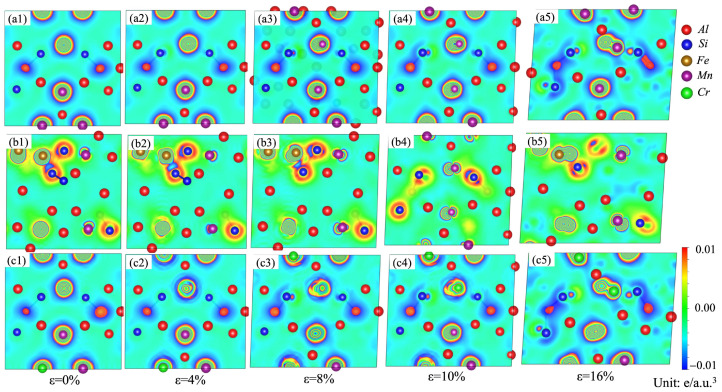
Charge-density difference on the (010) plane for the (**a1**–**a5**) α-AlMnSi; (**b1**–**b5**) α-AlFeMnSi and (**c1**–**c5**) α-AlFeMnCrSi phases during tensile strain.

The charge-density differences on the (010) plane for the α-AlMnSi, α-AlFeMnSi and α-AlFeMnCrSi phases with different tensile strains are shown in Figure 12. At the first stage, the high-density contours around the Mn, Fe and Cr atoms are circular, meaning that there is a uniform distribution of electrons in these phases. With the increase in strain, the distribution of electrons tends to become elliptical. This is evident from Figure 12(a1–a5), where charges around the Mn atoms in the α-AlMnSi phase are redistributed during the tensile process, leading to an increased tensile strength of the α-AlMnSi phase with higher tensile strain. The stronger Si-Fe bonds and the more obvious charge redistribution between Fe and Si atoms in the α-AlFeMnSi phase result in greater tensile stress (Figure 12(b1–b5)). For the α-AlFeMnCrSi phase, the charge redistribution around the Cr atoms is the key factor of the improved tensile strength of the α-AlFeMnCrSi phase (Figure 12(c1–c5)).

## 4. Conclusions

In this paper, the crystal structure, thermal stability, electronic structure and thermophysical and mechanical properties of the multicomponent α-AlFeMnSi and α-AlFeMnCrSi phases were investigated by experimental studies and first-principles calculation. Conclusions can be drawn as follows:

(1) The substitution of Mn atoms by Fe and Cr in α-AlMnSi are confirmed by structural determination, composition analysis and formation enthalpy calculation; Fe and Cr prefer to occupy Mn-12j sites in the α-AlMnSi phase to form the α-AlFeMnCrSi phase.
(2) The energetically favored structures of α-AlFeMnSi and α-AlFeMnCrSi show an improved stability, mechanical modulus and hardness, both in calculation and experimental results, which is attributed to the stronger bonding strength of Si-Fe and Si-Cr. The stronger chemical bonds of Si-Fe and Si-Cr lead to a decrease in the thermal expansion coefficient and an increment in the stacking fault energy and ideal strength.
(3) The mechanical modulus and hardness of the α-AlMnSi, α-AlFeMnSi and α-AlFeMnCrSi phases decrease with increasing temperature, which is validated by nanoindentation. The thermal expansion coefficient exhibits the opposite trend.

## Figures and Tables

**Table 1:** The chemical compositions of the as-prepared alloys (wt.%).

	Si	Fe	Mn	Cr	Al
A1	6.99	0.49	1.56	0.01	Bal.
A2	7.03	0.54	1.49	0.61	Bal.

**Table 2:** Variation of Young’s modulus (*E*) and hardness values (*H*) (in GPa) of the α-AlFeMnSi and α-AlFeMnCrSi phases at elevated temperatures.

	α-AlFeMnSi		α-AlFeMnCrSi	
	*E* (GPa)	*H* (GPa)	*E* (GPa)	*H* (GPa)
300 K	215.9	14.2	224.8	16.98
400 K	205.2	13.3	218.5	15.84
500 K	199.1	12.8	210.6	14.7
600 K	192.6	11.9	201.5	13.1

**Table 3:** Structural information, lattice parameters (Å), cell volume (Å^3^) and formation enthalpy *E_f_* (eV/atom) at 0 K for Cr or Fe occupying the Mn sites in the α-AlMnSi phase.

Crystal Structures	Atom Coordinates	*a*	*b*	*c*	*V* (Å^3^)	*E_f_*
α-AlMnSi	-	12.64	12.60	12.69	2021.1	−1.292
α-AlFe_Mn-12j_MnSi	Mn-12j (0.328, 0.197, 0)	12.71	12.70	12.70	2050.0	−2.166
α-AlFe_Mn-12k_MnSi	Mn-12k (0.18, 0.308, 0.5)	12.69	12.71	12.70	2048.4	−1.795
α-AlCr_Mn-12j_MnSi	Mn-12j (0.328, 0.197, 0)	12.69	12.71	12.71	2050.0	−2.502
α-AlCr_Mn-12k_MnSi	Mn-12k (0.18, 0.308, 0.5)	12.71	12.69	12.70	2048.4	−2.346
α-AlFeMnSi	-	12.65	12.62	12.71	2029.1	−2.722
α-AlFeMnCrSi	-	12.62	12.58	12.68	2013.1	−3.531

**Table 4:** Calculated elastic constants and modulus (in GPa) of α-AlMnSi, α-AlFeMnSi and α-AlFeMnCrSi phase at 0 K.

Species	*C*_11_	*C*_12_	*C*_13_	*C*_22_	*C*_23_	*C*_33_	*C*_44_	*C*_55_	*C*_66_	$\bar{C}$ **_11_**	$\bar{C}$ **_12_**	$\bar{C}$ **_44_**
α-AlMnSi	296.4	94	105.2	210.7	102.1	129.2	132.2	50.1	29.2	212.1	100.4	70.5
α-AlFeMnSi	312.7	103.6	113.2	220.6	105.4	120.6	164.4	52.6	31.3	218	107.4	67.1
α-AlFeMnCrSi	325.5	106.5	112.2	223.4	105.9	133.8	168.7	57.9	38.6	227.6	108.2	88.4

**Table 5:** Calculated mean bond lengths (Å) of α-AlMnSi, α-AlFeMnSi and α-AlFeMnCrSi phase at different temperatures.

Phases	*x*-*y* Bonds	Mean Bond Lengths (Å) at Different Temperatures				
		0 K	200 K	400 K	600 K	800 K
α-AlMnSi	Al-Si	2.83	2.85	2.86	2.87	2.87
	Al-Mn	2.67	2.68	2.68	2.68	2.69
	Si-Mn	2.59	2.60	2.59	2.60	2.63
α-AlFeMnSi	Al-Si	2.85	2.86	2.88	2.90	2.92
	Al-Mn	2.68	2.70	2.72	2.73	2.75
	Si-Mn	2.60	2.60	2.61	2.64	2.67
	Al-Fe	2.75	2.79	2.78	2.80	2.79
	Si-Fe	2.52	2.53	2.55	2.57	2.59
α-AlFeMnCrSi	Al-Si	2.83	2.85	2.86	2.87	2.88
	Al-Mn	2.68	2.69	2.70	2.71	2.71
	Si-Mn	2.65	2.66	2.66	2.70	2.71
	Al-Fe	2.73	2.75	2.78	2.78	2.79
	Si-Fe	2.65	2.65	2.65	2.66	2.67
	Al-Cr	2.65	2.66	2.67	2.71	2.71
	Si-Cr	2.56	2.58	2.58	2.64	2.65

## Data Availability

The raw/processed data required to reproduce these findings cannot be shared at this time as the data also form part of an ongoing study.
